# 5-Bromo-2-hy­droxy­benzaldehyde thio­semicarbazone

**DOI:** 10.1107/S1600536810043357

**Published:** 2010-10-31

**Authors:** Hadi Kargar, Reza Kia, Mehmet Akkurt, Orhan Büyükgüngör

**Affiliations:** a Department of Chemistry, School of Science, Payame Noor University (PNU), Ardakan, Yazd, Iran; b Department of Chemistry, Science and Research Branch, Islamic Azad University, Tehran, Iran; c Department of Physics, Faculty of Arts and Sciences, Erciyes University, 38039 Kayseri, Turkey; d Department of Physics, Faculty of Arts and Sciences, Ondokuz Mayıs University, 55139 Samsun, Turkey

## Abstract

The mol­ecule of the title compound, C_8_H_8_BrN_3_OS, is close to being planar, with maximum deviations of −0.127 (3) and 0.135 (5) Å for the N atoms of the –NH– and NH_2_– groups, respectively. Intra­molecular N—H⋯N and O—H⋯N hydrogen bonds to the same acceptor N atom generate *S*(5) and *S*(6) ring motifs. In the crystal structure, mol­ecules are connected into [010] chains by pairs of N—H⋯S hydrogen bonds with *R*_2_^2^(8) graph-set motifs. The crystal used for data collection was found to be an inversion twin.

## Related literature

For background on the biological activities and pharmaceutical properties of thio­semicarbazones and their derivatives, see: Casas *et al.* (2000 ▶); Ferrari *et al.* (2000 ▶); Maccioni *et al.* (2003 ▶). For bond-length data, see: Allen *et al.* (1987 ▶). For hydrogen-bond motifs, see: Bernstein *et al.* (1995 ▶).
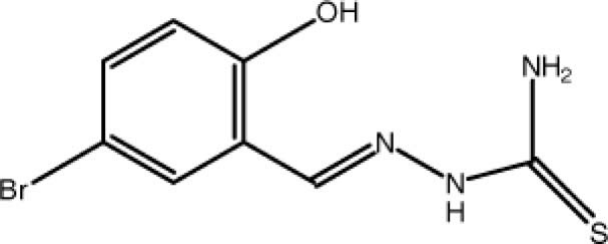


## Experimental

### 

#### Crystal data

C_8_H_8_BrN_3_OS
*M* *_r_* = 274.14
Orthorhombic, 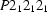

*a* = 4.4564 (2) Å
*b* = 8.3515 (3) Å
*c* = 27.7153 (14) Å
*V* = 1031.50 (8) Å^3^
*Z* = 4
Mo *K*α radiation
μ = 4.16 mm^−1^
*T* = 296 K
0.13 × 0.09 × 0.05 mm

#### Data collection

Stoe IPDS II diffractometer
Absorption correction: integration (*X-RED32*; Stoe & Cie, 2002 ▶) *T*_min_ = 0.614, *T*_max_ = 0.819
9532 measured reflections
1934 independent reflections
1782 reflections with *I* > 2σ(*I*)
*R*_int_ = 0.037

#### Refinement

*R*[*F* ^2^ > 2σ(*F* ^2^)] = 0.030
*wR*(*F* ^2^) = 0.063
*S* = 1.04
1934 reflections
144 parameters
4 restraints
H atoms treated by a mixture of independent and constrained refinement
Δρ_max_ = 0.54 e Å^−3^
Δρ_min_ = −0.29 e Å^−3^
Absolute structure: Flack (1983 ▶), with 744 Freidel pairs
Flack parameter: 0.477 (11)

### 

Data collection: *X-AREA* (Stoe & Cie, 2002 ▶); cell refinement: *X-AREA*; data reduction: *X-RED32* (Stoe & Cie, 2002 ▶); program(s) used to solve structure: *SIR97* (Altomare *et al.*, 1999 ▶); program(s) used to refine structure: *SHELXL97* (Sheldrick, 2008 ▶); molecular graphics: *ORTEP-3* (Farrugia, 1997 ▶); software used to prepare material for publication: *WinGX* (Farrugia, 1999 ▶).

## Supplementary Material

Crystal structure: contains datablocks global, I. DOI: 10.1107/S1600536810043357/hb5704sup1.cif

Structure factors: contains datablocks I. DOI: 10.1107/S1600536810043357/hb5704Isup2.hkl

Additional supplementary materials:  crystallographic information; 3D view; checkCIF report

## Figures and Tables

**Table 1:** Hydrogen-bond geometry (Å, °)

*D*—H⋯*A*	*D*—H	H⋯*A*	*D*⋯*A*	*D*—H⋯*A*
O1—HO1⋯N1	0.81 (3)	1.97 (4)	2.685 (4)	149 (5)
N3—HN3⋯N1	0.87 (4)	2.30 (4)	2.688 (5)	107 (3)
N2—HN1⋯S1^i^	0.84 (3)	2.55 (3)	3.373 (3)	168 (3)
N3—HN2⋯S1^ii^	0.86 (3)	2.50 (3)	3.363 (4)	176 (6)

Symmetry codes: (i) 
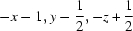
; (ii) 
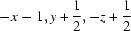
.

**Fig. 1.**
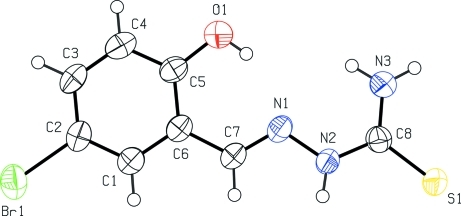
View of the title molecule, showing displacement ellipsoids for non-H atoms drawn at the 50% probability level.

**Fig. 2.**
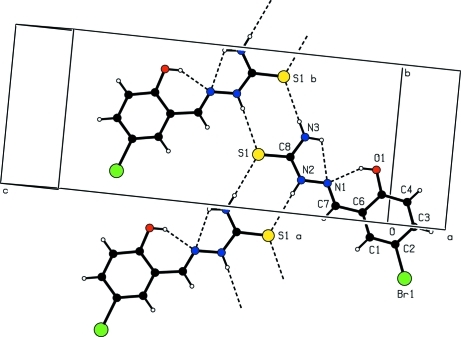
View of the hydrogen bonding interactions of (I), showing dimer formation by R22(8) ring motif within chains.

## supplementary crystallographic information

### Comment

Thiosemicarbazones constitute an important class of *N*,*S* donor ligands due to their propensity to react with a wide range of metals (Casas *et al.*, 2000). Thiosemicarbazones exhibit various biological activities and have therefore attracted considerable pharmaceutical interest (Maccioni *et al.*, 2003; Ferrari *et al.*, 2000). We here report the crystal structure of the title compound (I).

The title molecule (I) shown in Fig. 1 is almost planar with the maximum deviations of -0.127 (3) Å for N2 and 0.135 (5) Å for N3. All bond lengths and angles are normal (Allen *et al.*, 1987). In each independent molecule, there are intramolecular N—H···N and O—H···N hydrogen bonds, generating the *S*(5) and *S*(6) ring motifs, respectively (Table 1, Fig. 2).

In the crystal structure, adjacent molecules are linked by N—H···S hydrogen bonds, forming *R*_2_^2^(8) dimers (Bernstein *et al.*, 1995) (Table 1, Fig. 2).

### Experimental

A mixture of 5-bromosalicylalehyde (0.01 mol) and hydrazinecarbothioamide (0.01 mol) in 20 ml of ethanol was refluxed for about 2 h. On cooling, the solid separated was filtered and recrystallized from ethanol to yield colourless prisms of (I).

### Refinement

The H atoms of the O—H, N—H and NH_2_ groups were found from a difference Fourier map and their positional parameters were constrained [O1—H1 = 0.81 (3) and N2—HN1 = 0.84 (3), N3—HN2 = 0.86 (3) and N3—HN3 = 0.87 (4) Å]. Their isotropic displacement parameters were refined with *U*_iso_(H) = 1.2*U*_eq_(N) for the NH and NH_2_ groups and *U*_iso_(H) = 1.5*U*_eq_(O) for hydroxyl group. The remaining H atoms were placed in idealized positions and allowed to ride on their parent atoms, with C—H = 0.93 Å, and *U*_iso_(H) = 1.2*U*_eq_(C).

### Crystal data

**Table:**

C_8_H_8_BrN_3_OS	*F*(000) = 544
*M**_r_* = 274.14	*D*_x_ = 1.765 Mg m^−^^3^
Orthorhombic, *P*2_1_2_1_2_1_	Mo *K*α radiation, λ = 0.71073 Å
Hall symbol: P 2ac 2ab	Cell parameters from 17874 reflections
*a* = 4.4564 (2) Å	θ = 1.5–26.1°
*b* = 8.3515 (3) Å	µ = 4.16 mm^−^^1^
*c* = 27.7153 (14) Å	*T* = 296 K
*V* = 1031.50 (8) Å^3^	Prism, colourless
*Z* = 4	0.13 × 0.09 × 0.05 mm

### Data collection

**Table:**

Stoe IPDS II diffractometer	1934 independent reflections
Radiation source: sealed X-ray tube, 12 x 0.4 mm long-fine focus	1782 reflections with *I* > 2σ(*I*)
plane graphite	*R*_int_ = 0.037
Detector resolution: 6.67 pixels mm^-1^	θ_max_ = 25.6°, θ_min_ = 2.6°
ω scans	*h* = −5→5
Absorption correction: integration (*X-RED32*; Stoe & Cie, 2002)	*k* = −10→10
*T*_min_ = 0.614, *T*_max_ = 0.819	*l* = −33→33
9532 measured reflections	

### Refinement

**Table:**

Refinement on *F*^2^	Secondary atom site location: difference Fourier map
Least-squares matrix: full	Hydrogen site location: inferred from neighbouring sites
*R*[*F*^2^ > 2σ(*F*^2^)] = 0.030	H atoms treated by a mixture of independent and constrained refinement
*wR*(*F*^2^) = 0.063	*w* = 1/[σ^2^(*F*_o_^2^) + (0.0281*P*)^2^ + 0.5533*P*] where *P* = (*F*_o_^2^ + 2*F*_c_^2^)/3
*S* = 1.04	(Δ/σ)_max_ < 0.001
1934 reflections	Δρ_max_ = 0.54 e Å^−^^3^
144 parameters	Δρ_min_ = −0.29 e Å^−^^3^
4 restraints	Absolute structure: Flack (1983), with 744 Freidel pairs
Primary atom site location: structure-invariant direct methods	Flack parameter: 0.477 (11)

### Special details

**Table:**

Geometry. Bond distances, angles *etc*. have been calculated using the rounded fractional coordinates. All su's are estimated from the variances of the (full) variance-covariance matrix. The cell e.s.d.'s are taken into account in the estimation of distances, angles and torsion angles
Refinement. Refinement on *F*^2^ for ALL reflections except those flagged by the user for potential systematic errors. Weighted *R*-factors *wR* and all goodnesses of fit *S* are based on *F*^2^, conventional *R*-factors *R* are based on *F*, with *F* set to zero for negative *F*^2^. The observed criterion of *F*^2^ > σ(*F*^2^) is used only for calculating -*R*-factor-obs *etc*. and is not relevant to the choice of reflections for refinement. *R*-factors based on *F*^2^ are statistically about twice as large as those based on *F*, and *R*-factors based on ALL data will be even larger.

### Fractional atomic coordinates and isotropic or equivalent isotropic displacement parameters (Å^2^)

**Table:**

	*x*	*y*	*z*	*U*_iso_*/*U*_eq_	
Br1	0.80875 (10)	−0.32853 (5)	0.06313 (1)	0.0606 (1)	
S1	−0.5577 (2)	0.35791 (11)	0.26412 (3)	0.0517 (3)	
O1	0.2883 (6)	0.3331 (3)	0.08454 (10)	0.0596 (9)	
N1	−0.0227 (7)	0.2201 (3)	0.16022 (10)	0.0407 (9)	
N2	−0.2226 (8)	0.2237 (3)	0.19824 (9)	0.0408 (9)	
N3	−0.2703 (13)	0.4940 (4)	0.19194 (14)	0.0786 (16)	
C1	0.4380 (8)	−0.0920 (4)	0.10693 (12)	0.0416 (10)	
C2	0.6401 (7)	−0.1207 (4)	0.07038 (11)	0.0420 (10)	
C3	0.7260 (8)	0.0001 (5)	0.03956 (13)	0.0486 (11)	
C4	0.6033 (8)	0.1505 (5)	0.04464 (12)	0.0485 (11)	
C5	0.3981 (7)	0.1819 (5)	0.08111 (11)	0.0418 (10)	
C6	0.3119 (8)	0.0589 (4)	0.11293 (11)	0.0382 (9)	
C7	0.0966 (8)	0.0836 (4)	0.15148 (11)	0.0389 (10)	
C8	−0.3365 (8)	0.3610 (4)	0.21496 (11)	0.0416 (10)	
H1	0.38530	−0.17450	0.12780	0.0500*	
HN2	−0.323 (12)	0.587 (3)	0.2025 (16)	0.089 (16)*	
HN1	−0.260 (9)	0.135 (3)	0.2114 (12)	0.050 (11)*	
H3	0.86620	−0.01980	0.01540	0.0580*	
HO1	0.169 (8)	0.334 (6)	0.1064 (12)	0.071 (14)*	
H4	0.65840	0.23180	0.02350	0.0580*	
HN3	−0.144 (9)	0.486 (5)	0.1681 (12)	0.071 (14)*	
H7	0.04290	−0.00320	0.17060	0.0470*	

### Atomic displacement parameters (Å^2^)

**Table:**

	*U*^11^	*U*^22^	*U*^33^	*U*^12^	*U*^13^	*U*^23^
Br1	0.0606 (2)	0.0575 (2)	0.0638 (2)	0.0057 (2)	0.0074 (2)	−0.0153 (2)
S1	0.0657 (6)	0.0400 (5)	0.0495 (5)	−0.0007 (5)	0.0199 (4)	−0.0055 (4)
O1	0.0646 (16)	0.0493 (14)	0.0649 (15)	0.007 (2)	0.0180 (14)	0.0102 (14)
N1	0.0400 (16)	0.0458 (16)	0.0364 (13)	−0.0052 (13)	0.0067 (12)	−0.0039 (11)
N2	0.0436 (18)	0.0372 (14)	0.0415 (14)	−0.0028 (13)	0.0117 (13)	−0.0030 (11)
N3	0.122 (4)	0.0397 (17)	0.074 (2)	0.011 (2)	0.051 (3)	0.0063 (17)
C1	0.0408 (17)	0.0454 (19)	0.0387 (17)	−0.0065 (16)	0.0004 (15)	−0.0037 (15)
C2	0.0341 (18)	0.0532 (19)	0.0386 (16)	−0.0029 (14)	0.0002 (13)	−0.0098 (14)
C3	0.038 (2)	0.065 (2)	0.0428 (17)	−0.0032 (18)	0.0111 (15)	0.0010 (16)
C4	0.0427 (19)	0.062 (2)	0.0407 (16)	−0.0058 (19)	0.0012 (13)	0.0095 (17)
C5	0.0368 (17)	0.0475 (19)	0.0411 (15)	−0.0069 (17)	0.0005 (12)	0.0020 (15)
C6	0.0320 (14)	0.0477 (18)	0.0350 (15)	−0.0045 (17)	−0.0008 (15)	−0.0019 (13)
C7	0.0374 (19)	0.0462 (18)	0.0332 (15)	−0.0033 (15)	0.0027 (13)	−0.0010 (14)
C8	0.0465 (18)	0.0381 (18)	0.0402 (15)	−0.0006 (17)	0.0002 (15)	−0.0030 (13)

### Geometric parameters (Å, °)

**Table:**

Br1—C2	1.902 (3)	C1—C2	1.377 (5)
S1—C8	1.682 (3)	C1—C6	1.390 (5)
O1—C5	1.358 (5)	C2—C3	1.376 (5)
O1—HO1	0.81 (3)	C3—C4	1.377 (6)
N1—N2	1.380 (4)	C4—C5	1.388 (5)
N1—C7	1.281 (4)	C5—C6	1.407 (5)
N2—C8	1.337 (4)	C6—C7	1.451 (5)
N3—C8	1.315 (5)	C1—H1	0.9300
N2—HN1	0.84 (3)	C3—H3	0.9300
N3—HN2	0.86 (3)	C4—H4	0.9300
N3—HN3	0.87 (4)	C7—H7	0.9300
			
C5—O1—HO1	107 (4)	O1—C5—C4	117.7 (3)
N2—N1—C7	115.6 (3)	C5—C6—C7	122.6 (3)
N1—N2—C8	121.9 (3)	C1—C6—C5	118.5 (3)
C8—N2—HN1	122 (2)	C1—C6—C7	119.0 (3)
N1—N2—HN1	116 (2)	N1—C7—C6	122.7 (3)
HN2—N3—HN3	120 (4)	N2—C8—N3	118.1 (3)
C8—N3—HN2	122 (3)	S1—C8—N2	119.4 (3)
C8—N3—HN3	117 (3)	S1—C8—N3	122.5 (3)
C2—C1—C6	120.7 (3)	C2—C1—H1	120.00
Br1—C2—C3	119.6 (3)	C6—C1—H1	120.00
C1—C2—C3	120.7 (3)	C2—C3—H3	120.00
Br1—C2—C1	119.7 (2)	C4—C3—H3	120.00
C2—C3—C4	119.7 (3)	C3—C4—H4	120.00
C3—C4—C5	120.6 (4)	C5—C4—H4	120.00
C4—C5—C6	119.9 (4)	N1—C7—H7	119.00
O1—C5—C6	122.5 (3)	C6—C7—H7	119.00
			
C7—N1—N2—C8	172.0 (3)	C2—C3—C4—C5	1.2 (5)
N2—N1—C7—C6	−179.8 (3)	C3—C4—C5—O1	179.2 (3)
N1—N2—C8—S1	−175.2 (3)	C3—C4—C5—C6	−0.6 (5)
N1—N2—C8—N3	4.9 (5)	C4—C5—C6—C1	0.4 (5)
C2—C1—C6—C7	179.1 (3)	O1—C5—C6—C1	−179.5 (3)
C2—C1—C6—C5	−0.7 (5)	O1—C5—C6—C7	0.7 (5)
C6—C1—C2—Br1	180.0 (3)	C4—C5—C6—C7	−179.4 (3)
C6—C1—C2—C3	1.2 (5)	C1—C6—C7—N1	177.9 (3)
Br1—C2—C3—C4	179.8 (3)	C5—C6—C7—N1	−2.3 (5)
C1—C2—C3—C4	−1.5 (5)		

### Hydrogen-bond geometry (Å, °)

**Table:**

*D*—H···*A*	*D*—H	H···*A*	*D*···*A*	*D*—H···*A*
O1—HO1···N1	0.81 (3)	1.97 (4)	2.685 (4)	149 (5)
N3—HN3···N1	0.87 (4)	2.30 (4)	2.688 (5)	107 (3)
N2—HN1···S1^i^	0.84 (3)	2.55 (3)	3.373 (3)	168 (3)
N3—HN2···S1^ii^	0.86 (3)	2.50 (3)	3.363 (4)	176 (6)

Symmetry codes: (i) −*x*−1, *y*−1/2, −*z*+1/2; (ii) −*x*−1, *y*+1/2, −*z*+1/2.
